# Prevalence of intestinal parasitic infection in food handlers of **Iran**: A systematic review and meta‐analysis

**DOI:** 10.1002/vms3.590

**Published:** 2021-08-06

**Authors:** Khojasteh Sharifi‐Sarasiabi, Mehrgan Heydari‐Hengami, Azar Shokri, Saeed HosseyniTeshnizi

**Affiliations:** ^1^ Infectious and Tropical Diseases Research Center Hormozgan University of Medical Sciences Bandar Abbas Iran; ^2^ School of Medicine Hormozgan University of Medical Sciences Bandar Abbas Iran; ^3^ Vector‐borne Diseases Research Center North Khorasn University of Medical Sciences Bojnurd Iran

**Keywords:** food handlers, intestinal parasites, Iran, meta‐analysis, systematic review

## Abstract

Food handlers regardless of whether preparing or serving food, play key roles in the transmission of food‐borne infections. This study aimed to evaluate the prevalence of intestinal parasitic infections in food handlers in Iran. In the present study, a comprehensive literature search was carried out in electronic databases, including PubMed, Scopus, Google Scholar, Science Direct, Magiran, Scientific Information Database (SID), Iran Medex and Iran Doc, to identify all the published studies from 2000 to 31st April 2019. A total of 25 articles from different regions of Iran were identified and fulfilled our eligibility criteria. Totally, 140,447 cases were examined and 1163 cases were infected with intestinal parasites. Of all cases, 19,516 were male and 5901 were female with 1163 and 652 infected cases, respectively. The overall prevalence of intestinal parasitic infections was evaluated 14.0% [95% CI: 11.0‐17.0%]. It is revealed that protozoan, such as *Giardia lamblia*, with prevalence of 41.0% [95% CI: 25.0‐59.0%], *Blastosystis hominis* with 28.0% [95% CI: 15.0‐44.0%] and *Entamoeba coli* with 22.0% [95% CI: 16.0‐29.0%] had the highest prevalence while, *Dientamoeba fragilis* 5.0% [95% CI: 4.0‐7.0%], *Iodamoeba bütschlii* 5.0% [95% CI: 2.0‐8.0%], *Chilomastix mesnili* 5.0% [95% CI: 2.0‐9.0%] and *Endolimax nana* with 3.0% [95% CI: 1.0‐7.0%], were less prevalent. Infection with *Ascaris lumbricoides*7.0% [95% CI: 0.0‐29.0%] was more prevalent helminth followed with *Enterobius vermicularis* 3.0% [95% CI: 1.0‐5.0%], *Hymenolepis nana* 2.0% [95% CI: 1.0‐3.0%], *Taenia* spp. 2.0% [95% CI: 0.0‐7.0%] and *Trichuris trichiura* 1.0% [95% CI: 0.0‐1.0%]. The high prevalence of commensal parasites, such as *Entamoeba coli*, which does not need cure is indicating the importance of personal hygiene in food handlers.

Our results revealed the high prevalence of intestinal parasitic infection in food handlers in Iran. Monitoring programs to prevent and controlling of transmission to individuals are needed.

## Graphical abstract

Food handlers regardless of whether preparing or serving food, play key roles in transmission of food‐borne infections. This study aimed to evaluate the prevalence of intestinal parasitic infections in food handlers.

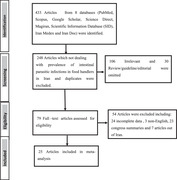

## 1 INTRODUCTION

Intestinal parasitic infections are widespread in the world and transmitting directly or indirectly among populations (FeizHadad et al., 2017). In some cases, carriers without any symptoms of the disease are the main source of infection especially if they work as food handlers. Given the high prevalence of 48.4 million cases of parasitic infections in the world, this fact is not reality. The importance of this issue emerges when those people work as food handlers and do not care about personal hygiene (Saki et al., 2012; Torgerson et al., 2015).

Although people are in constant contact with environmental pathogens, including parasites, they are not affected seriously since immunity is important in disease aetiology. Despite the good toleration of parasitic infection in healthiest individuals, some people are vulnerable to parasites (FeizHadad et al., 2017). The importance of parasitic infection is highlighted when the infected individual plays a major role in food handling or food industries.

Iran is a suitable region for most parasitesˊ growth and distribution due to the geographic, socioeconomic and behavioural conditions. Serious efforts to control parasitic infection have resulted in a burden decrease of parasitic infections, but contamination with intestinal parasites is still a concern for health‐care services (Kusolsuk et al., 2011). Using animal and human faeces as fertilizers for agriculture and vegetable gardens, climatic conditions, traditions, and customs are considered the main reasons for the incidence of parasitic infections in some parts of the country. Direct transmission from person to person is another factor that complicates the parasite control programs. This kind of parasite transmission is markedly important in food handlers and particularly in oral‐faecal parasites such as *Giardia lamblia* (*G. lamblia*)*, Hymenolepis nana* (*H. nana*) and *Enterobius vermicularis (E. vermicularis)* (Kusolsuk et al., 2011; Kheirandish et al., 2014). If food handlers do not care about personal hygiene, they can contaminate dishes, salads and other food materials which finally results in the contamination of the customers (Koohsar et al., 2012).

Studies on transmitted parasites by food handlers indicate that *Entamoeba coli* (*E. coli*) is the most common non‐pathogenic protozoa indicating a contamination with faecal materials and poor hygiene (Kassani et al., 2015). Also, zoonotic nature of some parasites, such as *Entamoeba histolytica (E. histolytica), Cryptosporidium parvum (C. parvum), H. nana, Taenia saginata (T. saginata), Giardia lamblia, Iodamoeba butschlii (I. butschlii), Chilomastix mesnili (C. mesnili), Endolimax nana (E. nana) and Entamoeba coli (E. coli)*, makes the control programs challengeable. Among all mentioned zoonotic parasites, some are more important and cause more morbidities, including *E. histolytica, C. parvum, T. saginata* and *G. lamblia* and need more attention from both humans and animals. Although, there was a doubt about the pathogenic nature of some protozoan, such as *Blastocystis hominis (B. hominis)*, in humans at present it is proven that they are associated with diarrhoea (Motazedian et al., 2016). Several studies have been conducted in different parts of the world regarding the prevalence of intestinal parasites in food handlers (Acilel et al., 2008; Abd Al‐Muhsin AL‐Khayat et al., 2017; Esparar et al., 2004; Kusolsuk et al., 2011; Wali et al., 2017). In this study, we performed a systematic review and meta‐analysis to find out the pooled estimate of the prevalence of intestinal parasites, such as *G. lamblia, E. coli*, *B. hominis* and *H. nana*, in food handlers, so the health‐care officials discovered the routes to prevent and control the disease transmitted by parasites and also, the best and most practical method used in conducting experiments to achieve the best results.

## 2 MATERIALS AND METHODS

This systematic review and meta‐analysis was conducted based on the guidelines of Preferred Reporting Items for Systematic Reviews and Meta‐Analyses (PRISMA) statement. The PROSPERO registration number is: CRD42019123662

### 2.1 Literature search and search strategy

**FIGURE 1:**
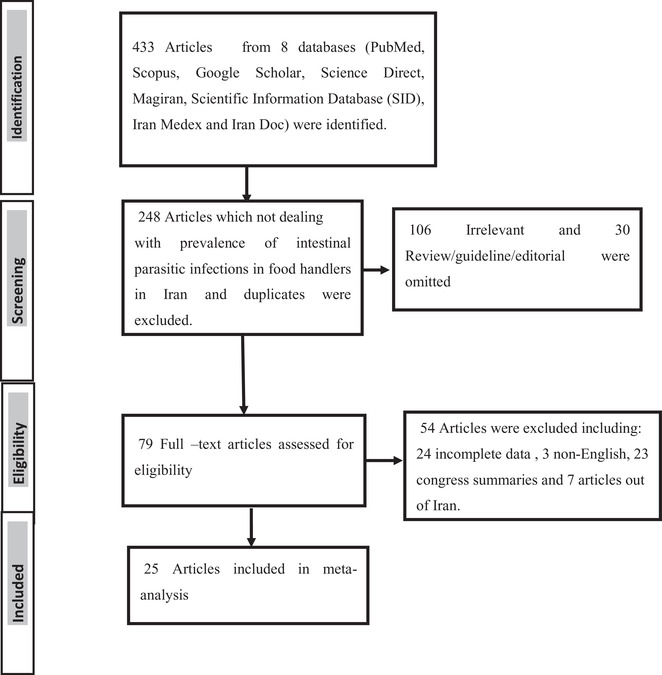
PRISMA flowchart describing the study design process

In this meta‐analysis, a comprehensive literature search was carried out in electronic databases, including PubMed, Scopus, Google Scholar, Science Direct, Magiran, Scientific Information Database (SID), Iran Medex, and Iran Doc, to identify all the published studies from 2000 to 31st April 2019. Duplicates and studies out of Iran were excluded. All original descriptive studies (designated as cross‐sectional) about intestinal parasites in food handlers were concerned. The process is shown in Figure 1. The search was performed using terms: ‘intestinal parasites’, ‘parasitic infection’, ‘parasitic diseases’, ‘parasite’, ‘food handlers’, ‘prevalence’, alone or in combination, both in Persian and English languages.

### 2.2 Data collection

In the initial search of collected bibliographic references, 433 articles were found. After removing duplicated, irrelevant studies and studies out of Iran, finally, 25 articles with epidemiological parameters of interest fulfilled the inclusion criteria. Those articles reporting the prevalence of intestinal parasitic infections in food handlers in Iran were included to our study (Table 1).

**TABLE 1:** Baseline characteristics of included studies

Ref	Author	Province	City	N. sample	N. positive	Infection rate (%)	Age group with highest infection	(%) Infection in age group	Male	Female	Laboratory diagnostic technique
1	Balarak et al. (2016)	East Azarbaijan	Tabriz	4612	156	3.73	20‐40	55.8	3966	646	Formalin ether
2	Fallahizadeh et al. (2017)	Khozestan	Shush county	15132	778	5.14			349	429	Direct smear
3	Garedaghi et al. (2014)	East Azarbaijan	Tabriz	100	72	72			ND	ND	Direct smear, formalin ether
4	Hatami et al. (2018)	Tehran	Tehran	4072	271	6.7		33.3	3122	950	Direct smear, formalin ether
5	Heydari Hengami et al. (2018)	Hormozgan	Bandarabbas	800	279	34.9	40‐49	43.1	625	175	Direct smear, formalin ether, staining
6	Saki et al. (2012)	Khuzestan	Khuzestan	62007	20580	33.1			ND	ND	Direct smear, formalin ether, staining
7	Kheirandish et al. (2014)	Lorestan	Khorramabad	210	19	9	20‐40	31	184	26	Direct smear, formalin ether, staining
8	Kheirandish et al. (2011)	Lorestan	Khorramabad	816	96	11.9			ND	ND	Direct smear, formalin ether, staining
9	Mohammadzadeh et al. (2018)	East Azarbaijan	Tabriz	87	16	18.4	>50	43.21	81	6	Direct smear, formalin ether, staining
10	Motazedian et al. (2015)	Fars	shiraz	1021	105	10.4	21‐30	48.1	577	444	Direct smear, formalin ether
11	Neghab et al. (2006)	Shiraz	Shiraz	39	23	59.4			37	2	Direct smear, formalin ether
12	Sharif et al. (2015)	Mazandaran	Sari	1041	161		30–39	84	620	421	
13	Amiri et al. (2013)	Khorasan	Shahroud	801	75	16.2		35.7	535	266	
14	Khazan et al. (2014)	Mazandaran	Gonbad e kavus	100		1			ND	ND	Direct smear, formalin ether
15	Balarak et al. (2014)	Tehran	Qom	2925	112	3.8	20‐40	50.8	2614	311	Direct smear
16	Dargahi et al. (2016)	Tehran	Tehran	109	69	63.3			ND	ND	Direct smear, formalin ether
17	Asadi et al. (2011)	Khorasan	Neishabour	8142	424	5.2			ND	ND	Direct smear
18	Davami et al. (2006)	Markazi	Arak	460	201	43.7	13‐50	ND	455	5	Formalin ether
19	Salary et al. (2013)	Kerman	Kerman	7748					5318	2430	Direct smear
20	Fallah et al. (2004)	Hamadan	Hamadan	938	713	76			ND	ND	Direct smear, formalin ether
21	Koohsar et al. (2012)	Golestan	Gorgan	500	30	6	51‐60	11.8	398	102	Direct smear, flotation
22	Haraty Nejad Torbati et al. (2011)	Khorasan	Rashtkhar	9001	673	7.5	ND	ND	ND	ND	Direct smear
23	Safi et al. (2012)	Ahvaz	Ahvaz	14614	1693	10.1	ND	ND	ND	ND	Direct smear, formalin ether
24	Safi et al. (2013)	Ahvaz	Ahvaz	12444	632	4.5	ND	ND	ND	ND	Direct smear, formalin ether
25	Babaei pouya et al. (2018)	Azarbaijan	Ardabil	1000	26	3.1	31‐40		884	116	Direct smear, formalin ether

ND, Not defined.

### 2.3 Data extraction

Two authors screened the titles, abstracts and full text of literatures, independently. Any disagreements between two reviewers were resolved by discussion among researchers. Extracted data included first author name, the year of publication, prevalence rate, demographic information (age and gender), geographical region of study, diagnostic test, sample size (number of examined people), and the number of infected cases (Table 1).

### 2.4 Quality of study

To assess the quality of observational studies included in this meta‐analysis using a checklist as in Table 1. It contains 12 items with scores ‘Yes = 1’ and ‘No = 0’. The sum of scores is 0 to 12 and for including study in meta‐analysis a quality score of at least 8 is required.

### 2.5 Statistical analysis

After extracting the sample size and the number of positive infections for each study, the proportion of infection and standard error (SE) were computed. Before estimating pooled effect size, sensitivity analysis was used to explore the effect of each study on pooled effect size. Heterogeneity among studies assessed using both *Q*‐test which is suggested by the Cochrane Handbook (*p* < 0.1 as substantial heterogeneity) and *I*‐square index *I* ^2^ < 50%, as substantial heterogeneity). If we found substantial heterogeneity, sub‐group meta‐analysis (fixed or random effect model) was performed to compute the pooled prevalence of infection based on a characteristic such as sex, country, education, pathogenicity and parasite species. In addition to meta‐regression examined to find the source of heterogeneity. To detect sources of heterogeneity, we performed meta‐regression on publish year and sample size of studies.

To evaluate publication bias, we aided a funnel plot and egger's test as a statistical test (*p* < 0.1 as significant). If we detected a substantial publication bias, the trim and fill method was applied to estimate and adjust for the number of missing studies (due to publication bias) in a meta‐analysis (Ebrahim, 2006). All statistical analysis was performed by using Stata/MP software (version 14.0, College Station, TX, USA).

## 3 RESULTS

Among all searched databases (eight databases) and unpublished data from 2000 to 2019 (19 years), 25 articles were eligible to include in this systematic review and meta‐analysis. The literature searches and selection process are shown in Figure 1. Totally 1,40,447 cases were examined. As all studies did not define the gender of studied cases, in studies that defined the gender of participants, a number of 19,516 cases were male and 5901 cases were female with 1163 (13.0%) infected cases in males and 652 (8.0%) infected in females, respectively (Table 1). There was a significant difference between infection among males 13.0% (10.0‐15.0%) and females 8.0% (5.0‐11.0%) (*p* = 0.027) (Figure 5).

**FIGURE 2:**
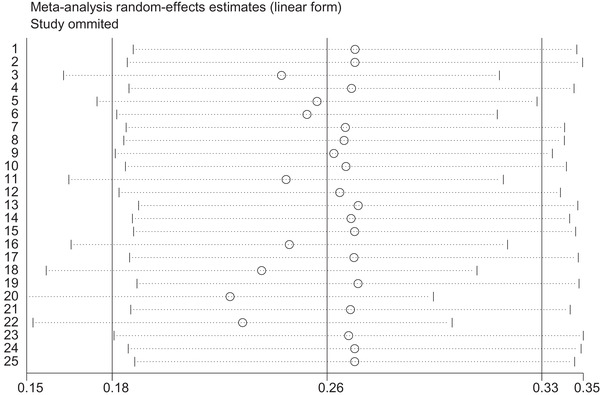
Sensitivity analysis to assess effect of each study on pooled effect size by omitting each study

To evaluate the effect of each study on the pooled estimate of prevalence, by repeating the meta‐analysis after omitting each study, the sensitivity of studies was depicted in Figure 2. All effect sizes of 25 studies were located in 95% confidence interval (95% CI). Therefore, none of the studies substantially affected the pooled prevalence of intestinal infection and we can include all studies in the meta‐analysis (Figure 2).

The results of Egger's test showed that there is no evidence of publication bias among studies on species of the parasite (*p* > 0.1). Also, there were not enough studies for assessing publication bias for *D. fragilis* and *T. trichiura* (Table 2).

**TABLE 2:** Comparison of the pooled frequency of infection among four parasite species

Characteristics	Levels	Sample	Prevalence (95% CI)	*I^2^* (%)	*p*
Gender	Male	14	29.0 (9.0‐38.0)	97.7	0.39
	Female	11	24.0 (18.0‐42.0)	89.2	
Age		25	22.0 (14.0‐32.0)	99.7	0.65
		7	29.0 (6.0‐60.0)	99.2	
		13	24.0 (9.0‐43.0)	99.1	

^*^The sample size was small for estimated pooled prevalence.

**FIGURE 3:**
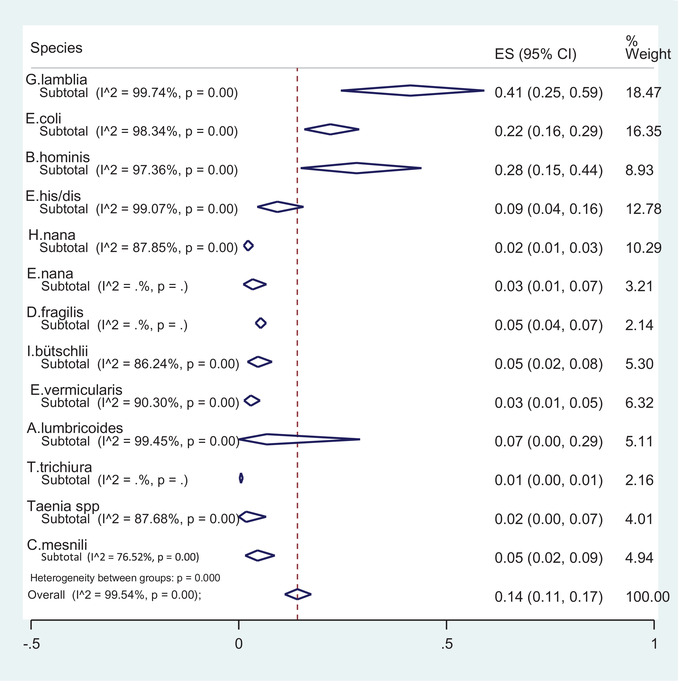
The forest plot of Intestinal parasites in food handlers in Iran

The overall prevalence of intestinal parasitic infections in food handlers in Iran was evaluated 14.0% (95% CI: 11.0‐17.0%). According to the results of sub‐group analysis, *G. lamblia*, with prevalence of 41.0% (95% CI: 25.0‐59.0%), *B. hominis* with 28.0% (95% CI: 15.0‐44.0%) and *E. coli* with 22.0% (95% CI: 16.0‐29.0%), had the highest prevalence, respectively. Also, other species had the prevalence between 1.0% (*T. trichiura*) to 9.0% (*E. histolytica/dispar*) (Figure 3).

The sub‐group analysis for intestinal protozoan parasites revealed the prevalence of *D. fragilis* 5.0% [95% CI: 4.0‐7.0%], *I. bütschlii* 5.0% [95% CI: 2.0%‐8.0%], *C. mesnili* 5.0% (95% CI: 2.0‐9.0%) and *E. nana* 3.0% (95% CI: 1.0‐7.0%). The results for intestinal helminthic infections showed that *A. lumbricoides* with prevalence of 7.0% (95% CI: 0.0‐29.0%) had the highest prevalence and then *E. vermicularis* with infection rate of 3.0% (95% CI: 1.0‐5.0%), *H. nana* with 2.0% (95% CI:1.0‐3.0%), *Taenia* spp. with 2.0% (95% CI: 0.0‐7.0%] and *T. trichiura* 1.0% [95% CI: 0.0‐1.0%] were the most prevalent intestinal helminthic infections (Figure 3). In this review, some of the parasites were non‐pathogenic (Tables 3,4).

**TABLE 3:** The results of examine publication bias for each parasite species

Species	N	bias	*p* *
*Giardia lamblia*	20	0.99	0.11
*Entamoeba coli*	17	−0.8	0.37
*Blastocystise hominis*	8	0.97	0.68
*Entamoeba hitolytica/dispar*	12	1.29	0.194
*Hymenolepis nana*	12	−0.15	0.37
*Endolimax nana*	3	−0.53	0.76
*Dientamoeba fragilis*	2	3.14	SS
*Iodamoeba butschlii*	5	−1.03	0.22
*Enterobius vermicularis*	5	0.35	0.78
*Ascaris lumbricoides*	7	−3.07	0.37
*Trichuris trichiura*	2	−1.86	SS
*Taenia saginata*	4	0.31	0.39
*Chilomastix mesnili*	5	0.69	0.15

SS, Small sample size.

* Results of Egger’ test.

**TABLE 4:** Intestinal parasitic infections in food handlers

					Type of Parasite: No(%)													
	Reference	No of Cases/No of infected Cases (%)	Job title	Most infected group No (%)	*E. coli*	*G. lambia*	*Blasto*	*Chilo*	*E. his*	*E. hart*	*Ioda*	*E. nana*	*D. fra*	*H. nana*	*As*	*Oxy*	*Trich*	Others
1	Fallah et al. (2004)	938/713 (76)	Food industry worker	ND	422 (45)	84 (9)		32 (3.4)	136 (14.5)	94 (10)	84 (9)	40 (4.3)	35 (3.7)	11 (1.1)	363 (38.7)	38 (20.3)	2 (0.2)	
2	Neghab et al. (2006)	39/24 (61.5)	Catering staff	ND	6 (2.3)	3 (1.2)	10 (3.9)	3 (1.2)						0				
3	Davami et al. (2006)	460/201 (43.7)	Food industry worker	Bakery workers	79 (17.2)	29 (6.3)								5 (1.1)				
4	Kheirandish et al. (2011)	816/97 (11.9)	Bakery workers	Bakery workers 97 (11.9%)	45 (5.5)	35 (3.7)	17 (2.1)							1 (0.1)				
5	Kohsar et al. (2011)	500/30 (6)	Food deliverers	Butchers 8(25%)	5 (1)	17 (3.4)								3 (0.6)				
6	Haraty Nejad Torbati et al. (2011)	729/55 (7.5)	Food industry worker	ND	47 (6.7)	376 (55.9)									3 (0.4)	47 (7)		201 (30)
7	Asadi et al. (2011)	8142/423 (5.2)	Food industry worker	ND		263 (3.2)			81 (1)							2 (0.25)		151 (1.9)
8	saki et al. (2012)	62007/4830 (7.8)	Food handlers	Food handlers	5643 (9.1)	2804 (4.52)	7019 (11.32)	˂(0.5)	865 (1.39)		3100 (5)			802 (1.29)	359 (0.57)		˂(0.5)	˂(0.5)
9	Safi et al. (2012)	14614/1693 (11.6)	Food industry worker	ND	128 (7.58)	1445 (85.35)			31 (1.83)					60 (3.54)		20 (1.71)		
10	Safi et al. (2013)	12444/632 (5.1)	Food industry worker	ND	33 (5.86)	510 (80.69)			20 (3.17)					46 (7.28)		19 (3.01)		
11	Salary et al. (2013)	7748/93 (1.2)	Food industry worker	Supermarket Owners (1.2)		96 (1.2)												
12	Garedaghi et al. (2014)	100 1 (1)	Restaurant workers	ND		(16.66)		(36.11)	(47.22)									
13	Amiri et al. (2014)	801/141 (17.6)	Food industry worker	ND	74 (9.2)	35 (4.4)		7(0.9)		12 (1.5)	1 (0.1)	1 (0.1)			1 (0.1)			1 (0.1)
14	Khazan et al. (2014)	100/1 (1)	Food sellerts	ND								1 (1)						
15	Kheirandish et al. (2014)	210/19 (9)	Food industry worker	ND	8 (4.3)	7 (2.9)	3 (1.4)							1 (0.5)				
16	Motazedian et al. (2015)	1021/106 (10.4)	Food handlers	Herbal sellers 25 (16)	40 (37.7)	27 (25.5)	40 (37.7)	5 (4.7)			7 (6.6)	1 (0.9)						
17	Balarak et al. (2015)	2925/112 (3.8)	Food industry worker	Restaurant 16 (14.5) Supermarket Workers 25 (22.3)	22 (19.6)	74 (66)								4 (3.6)	7 (6.3)			
18	Sharif et al. (2015)	1041 161/(15.5)	Food industry worker	Restaurant 38 (19.2) Fast food worker 33 (17.8)	25 (15.5)	86 (53.4)	29 (18)		9 (5.6)		5 (3.1)			30 (18.6)				5 (3.1)
19	Dargahi et al. (2016)	109/69 (63.3)	Restaurant workers	ND		69												
20	Balarak et al. (2016)	4612/172 (3.7)	Food industry worker	Supermarket workers 38 (22.1) Fast food worker 33 (19.2)	38 (22)	109 (63)			9 (5.2)					6 (3.5)	10 (5.8)			
22	Hatami et al. (2018)	4072 271 (6.6)	Food handlers	ND	72 (26.6)	148 (54.6)			21 (7.7)						19 (7)	1 (0.3)		11 (3.7)
23	Heydari Hengami et al. (2018)	800/279 (34.9)	Food handlers	Restaurant worker 58 (33.9) Supermarket worker 52 (34.7)	64 (22.9)	54 (19.4)	194 (69.5)		7 (2.2)		8 (2.9)	2 (0.4)	34 (12.2)	2 (0.4)				
24	Mohammadzadeh et al. (2018)	87/16 (18.4)	Food handlers	Chef 2 (66.7)	5 (19.2)		5 (19.2)	5 (19.2)				9 (34.6)		1 (3.8)	1 (3.8)			
25	Babaei pouya et al. (2018)	1000/31 (3.1)	Food handlers	Restaurant workers 12 (38.7)	8 (0.8)	14 (1.4)	6 (0.6)							2 (0.2)				1 (0.1)

ND, not defined; H. nana, Hymenolepis nana; *E.coli*, *Entamoeba coli*; *G.lamb*, *Giardia lambelia*; *Blasto*, *Blastocystis hominis*; *Chilo*, *Chilomastix mesnili*; *E.his*, *Enatamoeba histolytica/dispar*; *E.hart*, *Entamoeba hartmani*; *Ioda*, *Iodamoeba butschlii*; *E.nana*, *Endolimax nana*; *D.fra*, *Dientamoeba fragilis*.

*AS, Ascaris lumbericoides*; Oxy, *Oxyuris vermicularis*; Trich, *Trichuris trichiura*.

The highest rate of infection was found in owners of the school cafeterias with 28.0% followed by 11.50% in butchers and 10.20% among bakeries. The lowest infection rate was 1.70% in confectioners (Tables 3, 5). The results of meta‐regression showed that the prevalence of intestinal parasitic infection in food handlers has significantly decreased in recent years (*p* = 0.01). Also, our analysis revealed that sample size did not affect the prevalence of intestinal parasitic infection in food handlers (*p* = 0.68). To evaluate the effect of each study on the pooled prevalence, by meta‐analysis, the sensitivity of studies is shown in Figure 2. At the first level, a fixed‐effect meta‐analysis was performed on 25 included studies and results revealed considerable heterogeneity (*I*ˆ*^2 ^* = 99.40%, *p* < 0.001). In sub‐group analysis, a random effect model was performed on parasite species (Figure 3). All effect sizes of 25 studies were located with 95% interval confidence. Therefore, studies did not affect the pooled prevalence of intestinal infections in food handlers and we can include all studies in the meta‐analysis (Figure 2).

**TABLE 5:** Distribution of intestinal parasitic infection in different jobs

		Job title								
	Authors	Bakery No cases/Inf	Supermarket owner No cases/Inf	Restaurant/fast food workers No cases/Inf	Butcher No cases/Inf	Coffee shop owner No cases/Inf	Office servant No cases/Inf	Food Factory workers No cases/Inf	School cafeteria No cases/Inf	Confectioner No cases/Inf
1	Balarak et al. (2016)	274/9	880/38	821/24	95/4	229/33	889/13			
2	Fallahizadeh et al. (2017)									
3	Garedaghi et al. (2014)									
4	Hatami et al. (2018)									
5	Heydari Hengami et al. (2018)	81/44	150/52	233/80	33/9	86/23	161/48	56/23		
6	Saki et al. (2012)									
7	Kheirandish et al. (2014)									
8	Kheirandish et al. (2011)	816/97								
9	Mohammadzadeh et al. (2018)			87/16						
10	Motazedian et al. (2015)	28/3	125/15	244/27	48/6		80/3	163/21		46/2
11	Neghab et al. (2006)									
12	Sharif et al. (2015)	112/9		383/71	204/27	36/3			18/5	
13	Amiri (2014)									
14	Khazan et al. (2014)									
15	Balarak (2015)	172/6	533/25	954/36	48/3	207/14		623/9		130/5
16	Dargahi et al. (2016)									
17	Asadi (2011)									
18	Davami et al. (2006)									
19	Salary et al. (2013)		2256/35	1709/28		2161/21				673/7
20	Fallah (2005)									
21	Kohsar (2011)	123/3	181/12	92/8	8/6	20/3	13/0			63/2
22	Haraty Nejad Torbati et al. (2011)									
23	Safi (2012)									
24	Safi (2013)									
25	Babaei pouya (2018)	144/7	125/2	136/12	68/3					66/1

## 4 DISCUSSION

Food‐borne parasitic diseases are one of the main public‐health concerns all around the world which may lead to morbidity and mortality in developing countries (Simsek et al., 2009). The importance of hygienic food preparation and delivery reveals the importance of personal sanitation and education in food handlers. This group of people is involved in handling, storage, transportation, process and preparation of food on several levels for other peoples. This systematic review and meta‐analysis aimed to evaluate the prevalence of intestinal parasitic infections in food handlers in Iran during 19 years (from 2000 to 2019). The results of the meta‐analysis revealed the overall prevalence of intestinal parasitic infections was 14.0% [95% CI: 11.0‐17.0%] in food handlers in Iran. The results indicated poor health and inadequate personal hygiene in food handlers who are involved in food‐producing and food‐serving processes in Iran. The highest rate (72.0%) of infection was reported in a study carried out in East Azarbaijan by Garedaghi et al. (2014); Dargahi et al. (2016) who reported the rate of 59.4% in Tehran province. The lowest prevalence of infection (1.0%) was reported from Mazandaran province by Khazan et al. (2014) (Table 1). The sub‐group analysis revealed that *G. lamblia* with the prevalence of 41.0% [95% CI: 25.0‐59.0%], *B. hominis*, with 28% [95% CI: 15.0‐44.0%] and *E. coli* with 22.0% [95% CI: 16.0‐29.0%], had the highest prevalence among all intestinal parasites in food handlers in Iran. Although we know that *E. coli* is a non‐pathogenic parasite and the infection only reflects personal and public health condition but, it is considerable in persons who are working as food handlers.

The highest rate of infection (28.0%) was achieved in owners of school snack bars, where children took cooked food and snacks. The results may have a bias for a small sample size, but the important point in this regard is that 5 of 18 different school cafeteria owners were infected with intestinal parasites which are significant. This may have resulted from weak health controlling programs in schools. In a study carried out by Costa‐ Cruz et al. in Brazil, the researchers studied 20 schools for the evaluation of intestinal parasitic infections in school food handlers. They found that 49 of 104 (47.10%) of school food handlers were infected (Khazan et al., 2013). Comparing their findings with ours indicates the higher rate of infection in their studied subjects. The meta‐analysis revealed the high prevalence of intestinal parasitic infection in butchers (11.50%) and backers (10.20%). These two groups play an important role in public food health. Interestingly, the lowest prevalence of intestinal parasitic infection rate was observed among confectioners (1.70%). Although the sample size comprised 978 cases and relatively big, the results indicate appropriate personal hygiene in this group which is regularly monitored by the health‐care system.

Also, our meta‐analysis revealed the infection rate in males (13%) was significantly higher than females (8%) which may be resulted from a smaller sample size in females and less involvement of females in food‐handling processes than males in Iran. In some countries, the ratio of male to female was different from ours. In a study in Thailand in 2011, Kusolsuk et al., studied 219 females and 47 males. This has resulted from the great role of females in food preparing and handling in Thailand. The result of their study revealed that the infection rate in 273 food handlers was 10.30% which is higher than our results when compared with the infection rate of 14.0% in 1,40,447 subjects in our study. In contrast with our results, the most infecting cases were found with hookworms (70%) while our most prevalent helminthic infection was with *H. nana* worms (Kusolsuk et al., 2013). Their results revealed insufficient hygiene in food preparation and our results indicated inappropriate personal hygiene. Our meta‐analysis showed that the highest intestinal infection in food handlers was caused by protozoan parasites and the most frequent parasite (41.0%) was *G. lamblia* (Figure 3). These protozoa are among the most pathogenic parasites (Arora, 2015) which can cause acute or chronic diarrhoea with or without clinical signs. The parasite can be transmitted directly from infected persons to healthy individuals. Therefore, eradication and controlling this parasite is very difficult. It is estimated that 200 million people in Asia, Latin America and Africa suffering from giardiasis (Abd Al‐Muhsin AL‐Khayat et al., 2017). In a study carried out by Simsek et al. in 2009 in Turkey, intestinal parasitic infection was evaluated in 299 food handlers from Sanliurfa, Southeastern Anatolia. The results showed that 52.20% of food handlers were infected with intestinal parasites and most of them (26.80%) were infected with *G. lamblia*, followed by *A. lumbricoides* (10.70%) and *T. saginata* (10.0%). Also, 13.30% of them were infected with both *Staphylococcus aureus* and intestinal parasites. Unlike our results, the infection rate with *G. lamblia* in their study was higher.

**FIGURE 4:**
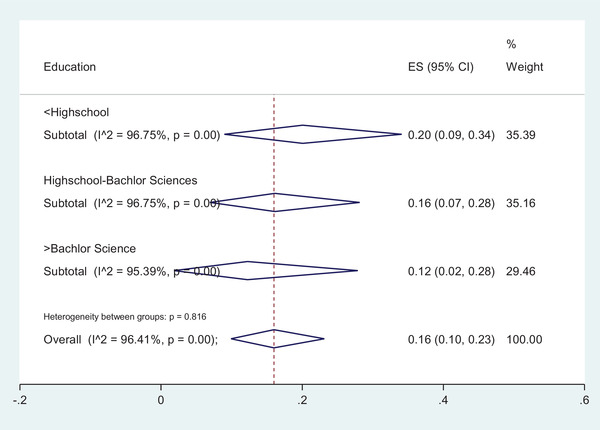
The forest plot of pooled prevalence of intestinal parasitic infection according to the educational levels

The meta‐analysis elucidated that the prevalence of intestinal parasitic infection in individuals with education level lower than high school, was 20.0% [95% CI:9.0‐34.0%] while in individuals with education level between high school to the bachelor of science level, was 16.0% [95% CI:7.0‐28.0%] and in cases with education higher than bachelor of science level was reduced to 12.0% [95% CI: 2.0‐28.0%] but, there found no statistically significant difference (*Z* = 0.41, *p* = 0.82) (Figure 4). Although the results indicated no association between intestinal parasitic infection and educational levels but, it seems that the infection rate in individuals with lower levels is higher than those with higher educational levels. It seems that food hygiene knowledge, attitudes and practices in food handlers play an important role in the prevention of food contamination with intestinal parasites. In a study designed by Acikel et al. in 2008, a total of 83 food handlers in the kitchen were evaluated with questionnaires for their information and behaviours before and after training. The results indicated a significant difference in behavioural practices, and the researchers concluded that education has an important impact on decreasing the infection rate in food handlers. Although the researchers studied the decreased bacterial density, it can be extended in parasitic infections too as the way of transmission is almost the same (Acikel et al., 2008). In a study by Kheirandish et al., in 2011, out of 816 bakery workers with health certificates, 630 individuals knew about intestinal parasitic infections and the ways of transmission but, 78 (12.30%) of them were infected with intestinal parasites. Also, 186 (22.80%) of this population had no knowledge in this regard and 19 (10.20%) individuals were infected among them. These researchers declared that 85% of intestinal parasitic infections were observed in people who did not attend hygiene training programs. This shows that training to upgrade personal information in parasite transmission is necessary for all food handlers. Also, training hygiene can affect the improvement of society's health (Kheirandish et al., 2011).

**FIGURE 5:**
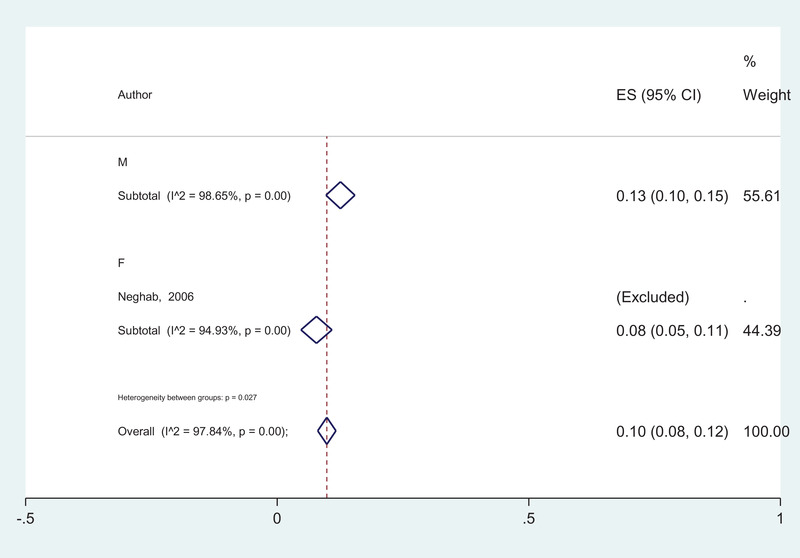
The forest plot of pooled prevalence of intestinal parasitic infection according to the gender

## 5 CONCLUSIONS

Our results revealed the high prevalence of intestinal parasitic infection in food handlers in Iran. This high prevalence is largely due to poor personal hygiene practice, poverty, lack of knowledge, insufficient environmental sanitation and inadequate health controlling services. Although the food industry workers, food handlers, and anyone who is connected with the production, handling, storage, transportation, preparation, or else, is obliged to undergo routine medical examinations including stool microscopy for intestinal parasitic infections (once every 6 months) but, it seems that they are not sufficient. It is advised that some strict rules such as obligation in filling the stool container in the lab should be added. Also, if infected food handler cases are identified, immediate decisions for the exclusion of the career up the resolving all symptoms or completion of further investigations should be made. Additional programs, including education for changing attitude about infectious diseases requires more consideration.

## CONFLICT OF INTEREST

The authors declare that they do not have any conflict of interest.

## ETHICS APPROVAL

No ethical approval was required as this is a review article with no original research data.

## AUTHOR CONTRIBUTION

K. S.S. and M. H.H. were involved in data gathering. A. S. was Project administration and Supervisor; involved in writing‐review & editing data and critical revise. S. H. T. was involved in methodology; data validation; formal data analysis and critical revise.

### PEER REVIEW

The peer review history for this article is available at https://publons.com/publon/10.1002/vms3.590

## Data Availability

The data that support the findings of this study are available from the corresponding author upon reasonable request.
